# Difference between the prevalence of symptoms of depression and anxiety in non-diabetic smokers and in patients with type 2 diabetes with and without nicotine dependence

**DOI:** 10.1186/1758-5996-4-39

**Published:** 2012-08-21

**Authors:** Simone Franco Osme, LudmillaDell’IsolaPelegriniMelo Ferreira, Mariana Tanus Jorge, Juliana de Souza Andréo, MariaLuizaMendonçaPereira Jorge, Rogério de Melo Costa Pinto, Miguel Tanús Jorge, Paulo Tannús Jorge

**Affiliations:** 1 Departamento de Clínica Médica, Federal University of Uberlandia, Uberlândia, MG, 38400-902, Brazil; 2 Institute of Psychology, Federal University of Uberlândia, Uberlândia, MG, Brazil; 3 Departament of Psychology, Clinical Hospital, Federal University of Uberlândia, Uberlândia, MG, Brazil; 4 Endocrinology Clinic, Clinical Hospital, Federal University of Uberlândia, Uberlândia, MG, Brazil; 5 Program in Health Sciences, Medicine College, Federal University of Uberlândia, Uberlândia, MG, Brazil

**Keywords:** Smoking, Nicotine dependence, Diabetes mellitus, Depression, Anxiety

## Abstract

**Background:**

Individuals with diabetes who are smokers have higher risks of cardiovascular disease, premature death, and microvascular complications. The present study aims to determine the prevalence of symptoms of depression and anxiety in smokers with type 2 diabetes mellitus (T2D) and to evaluate if the prevalence of symptoms of depression and anxiety differ between the three groups studied (patients with T2D who smoke; patients with T2D who do not smoke; smokers without T2D), and finally determine if the degree of nicotine dependence is related to symptoms of anxiety and depression in smokers (with or without T2D).

**Methods:**

Three study groups were formed: 46 T2D smokers (DS), 46 T2D non-smokers (D), and 46 smokers without diabetes (S), totaling 138 participants. Hospital Anxiety and Depression (HAD) scale and Fagerström Test were applied.

**Results:**

The prevalence of symptoms of depression and anxiety in smokers with T2D was 30.4% and 50%, respectively. There was no significant difference in the proportion of individuals with symptoms of anxiety (p = 0.072) or depression (p = 0.657) in the DS group compared to group D or S. Among male patients with T2D, the smokers had a higher prevalence of anxiety symptoms (19.6%) than non-smokers (4,3%) (p = 0,025). The prevalence of high nicotine dependence among smokers with and without T2D was 39.1% and 37.1%, respectively (p = 0.999). Fagerström scores showed no significant correlation with the scores obtained on the subscale of anxiety (p = 0,735) or depression (p = 0,364).

**Conclusions:**

The prevalence of depression and anxiety among smokers with and without diabetes and non-smokers T2D is similar. Among male individuals with T2D, the smokers have more symptoms of anxiety than the non-smokers. There is no difference in the prevalence of nicotine dependence among smokers with and without diabetes. The presence of symptoms of anxiety or depression is similar between patients who are dependent and not dependent on nicotine.

## Background

Growing evidence suggests an association between smoking and the development of type 2 diabetes (T2D) in adults [1,2]. A meta-analysis showed that smoking is associated with increased risk of developing T2D and that this phenomenon is dose-dependent [3]. According to Wen *et al.*, the addition of smoking habit makes the appearance of diabetes earlier by an average of 4.1 years [2].

According to the International Diabetes Federation, the estimated prevalence of diabetes in a population aged between 20 and 79 years is 8.3%, and 80% of those individuals live in low- and middle-income countries. Worldwide, approximately 366 million adults are diabetics, including 12.4 million in Brazil. T2D accounts for at least 90% of all cases of diabetes [4]. According to the World Health Organization, cigarette smoking is the leading preventable cause of premature death, and the tobacco epidemic kills nearly six million people per year [5]. A study in Brazil showed that the prevalence of smoking in Brazilian capitals varies from 12.9% in Aracajú to 25.2% in Porto Alegre [6]. Another national survey found that 17.4% of the sample uses tobacco daily and that 20.8% of smokers aged ≥ 35 years reported a desire to stop or reduce tobacco use [7]. This study found that daily consumption of tobacco in major Brazilian cities is significantly lower so far in the 2000s than at the end of the last century. In contrast, a study conducted in the U.S. found that the prevalence of smoking among adults with diabetes (type 1 or 2) in 1990 was 23.6% and remained stable through 2001 (23.2%) [8].

Smokers have increased risks of cardiovascular disease, premature death, and microvascular complications of diabetes [9]. Cigarette smoking increases the risks of nephropathy, retinopathy, diabetic neuropathy [which is more strongly associated with type 1 diabetes (T1D)], and macrovascular complications (more pronounced in T2D) [10].

The intimate relationship of smoking with some psychiatric disorders, especially depression and anxiety, has been demonstrated [11]. Collins *et al.*[12] even consider smoking to be a risk factor for higher scores of anxiety and depression. Breslau *et al.*[13] demonstrated in young adults positive associations between nicotine dependence and major depression, obsessive compulsive type disorders, phobias, and anxiety disorders, as well as alcohol and illicit drug use.

Few studies have evaluated smoking in subjects with diabetes. The study of Spangler *et al.*[14] is the only study whose primary objective was to study the psychological variables in smokers with diabetes. Those authors found higher levels of stress, depression, and negative affect (anxiety, guilt, hostility) in smokers than non-smoking T1D patients. No study aiming to assess depression and anxiety in T2D smokers was found in the literature. However, Lloyd *et al.*[15] found no significant difference in the prevalence of depression (HAD scale) among smokers and non-smokers when assessing symptoms of depression and anxiety in outpatients with T1D or T2D.

Most smokers, despite the intention to quit, are unsuccessful. The Fagerström Tolerance Questionnaire and its revised version Fagerström Test for Nicotine Dependence (FTND) were specifically developed to evaluate the physical dependence to nicotine [16]. Emotional problems such as depression and anxiety are common in T2D patients and may impair self-care and glycemic control [17]. The American Diabetes Association (ADA) emphasizes that the presence of psychiatric comorbidities such as depression are associated with a higher prevalence of smoking and increased risk of relapse after stopping smoking [18]. The Hospital Anxiety and Depression (HAD) scale is used to evaluate symptoms of anxiety and depression [19] and is considered a reliable indicator of depression in individuals with diabetes [20]. The ADA recommends that all smokers be warned about the additional risks that smoking causes and be advised to cease the habit, including alternative forms of treatment and, in special situations, an evaluation of the degree of nicotine dependence [9,18].

The symptoms of depression and anxiety are both associated with smoking [12,14,21] and diabetes [21-26]. So, subjects with diabetes and smokers have two risk factors, and thus it is possible that they are more likely to have symptoms of anxiety and depression. The presence of psychiatric disorders was associated with nicotine dependence [11,13]. Thus, it is also expected that smokers with T2D have a higher degree of nicotine dependence.

The present study aimed to determine the prevalence of symptoms of depression and anxiety and the degree of nicotine dependence in smokers diagnosed with T2D who were treated at the Endocrinology Clinic in the Clinics Hospital, Federal University of Uberlândia (HC-UFU). These prevalences were compared with those found among the T2D non-smokers and those of the smokers without diabetes in an attempt to clearly define the role of each variable in symptomatology. Thus, the degree of nicotine dependence was also compared between T2D smokers and non-diabetics smokers. Finally, this study also aimed determine if the degree of nicotine dependence is related to symptoms of anxiety and depression in smokers (with or without T2D).

## Methods

Between March and November 2009, T2D patients aged 30 and older were interviewed. They were contacted at the Endocrinology Clinic, HC-UFU. All T2D out-patients who went to the Endocrinology Clinic were asked if they did or did not smoke and if they accepted to participate in the study. If the answer was yes, they immediately filled out the informed consent. The pharmacist researcher of this study was responsible for interviewing the patients. All patients gave their written informed consent for participation.

The diagnosis of T2D was confirmed in the medical records based on ADA criteria.

Each T2D smoker (case) was paired with one non-smoking T2D patient (control 1) who was treated in the same Endocrinology Clinic. The non-diabetic smokers (control 2) were arranged, by convenience, in the ophthalmology, angiology and dermatology clinic of the same hospital.

Sex and age could be potential confounders since both have association with the prevalence of symptoms of depression and anxiety [11,21,25,27] and with nicotine dependence [28], then the controls were matched by sex and age, differing ± 3 years.

Patients with no history of diabetes, who were not taking oral hypoglycemic agents or insulin, and who had fasting glucose < 126 mg/dL in the last 6 months were considered non-diabetic. Those who met the above criteria, except for not having undergone this test in the last 6 months, were subjected to glucometry measured by capillary at the time of the interview. They were considered non-diabetic if blood glucose was < 140 mg/dL.

Both T2D and non-diabetic patients were considered to be smokers when they reported consumed at least one cigarette per day for the past 6 months. Individuals who had consumed ≤ 100 cigarettes throughout their lives were considered to be non-smokers. Former smokers who reported having quit smoking for at least 1 year were also considered to be non-smokers.

Three groups of patients were formed: patients diagnosed with T2D who were smokers (group DS), non-smoking T2D patients (group D), and non-diabetic smokers (group S).

Exclusion criteria were severe psychiatric disorders; systemic disease, such as cancer, acquired immunodeficiency syndrome, or leprosy, in the medical record; thyroid-stimulating hormone < 0.3 mIU/mL or > 10 mIU/mL; use of antidepressants or antipsychotics; and cognitive deficits or recent and significant personal problems that, based on the investigators’ evaluation, could interfere with their responses.

All patients were interviewed in person by the authors, who filled out a pre-prepared questionnaire with information about education, marital status, alcohol consumption, physical activity, family history and habits related to smoking, use of antihypertensives and anxiolytics, time of diagnosis of T2D, family history of disease, and the treatments currently used, such as diet and the use of oral hypoglycemic agents and/or insulin. The weight and height of patients were measured with mechanical anthropometric scales, and the body mass index (BMI) (kg/m^2^) was based on these measures. Cohabitation with a spouse or partner was considered a stable marital status.

The HAD scale, which has been validated in Brazil by Botega *et al.*[19], was used as tool to assess symptoms of anxiety and depression. This scale was chosen because insomnia, fatigue, and weight loss, which could also be symptoms of physical illness, do not appear on this scale, which is focused on symptoms of anhedonia [19]. The HAD scale was applied by two psychology students who were previously trained by a psychologist specialized in health psychology. A score ≥ 8 points on the subscale HAD-A or HAD-D was considered indicative of clinically significant symptoms of anxiety or depression, respectively.

The FTND, adapted to the Brazilian population of smokers, was used as a tool to assess the degree of nicotine dependence. A score ≥ 6 indicates high dependence, and a score ≤ 5 indicates medium or low nicotine dependency [29].

Comparisons between groups were performed using Student's *t*-test for variables with normal distribution (Lilliefors test) and the Mann–Whitney *U*-test for variables with non-normal distribution. Analysis of variance (normal distribution of waste) and the Kruskal-Wallis test (Dunn) as a non-parametric test were used to compare three groups. The *χ*^2^ test was used to evaluate the relationships between qualitative variables. To verify the existence of a correlation between the scores or other values, Pearson's correlation was used. By logistic regression, we obtained the odds ratios (ORs) of anxiety, depression, nicotine dependence, and other factors. For variables that showed statistical significance, we performed multiple logistic regression. We used the significance level of 5%. BioEstat 5.0 software was used for statistical analysis [30].

The present study was approved by the ethics committee of Federal University of Uberlândia (protocol: 042/09).

## Results

We recruited 187 patients, five of whom were unwilling to participate and 44 who met the exclusion criteria. We therefore evaluated 138 patients, with 46 in each group.

The groups DS, D, and S were similar in terms of average age (53.26 ± 11.09, 53.33 ± 11.42, and 52.65 ± 11.56 years, respectively, p = 0.952). Their mean weight was 75.05 ± 15.55, 81.10 ± 14.39, and 71.08 ± 17.70 kg, and the mean BMI was 28.37 ± 5.51, 30.78 ± 6.07 and 26.51 ± 5.21 kg/m^2^, respectively. The proportions of obese subjects (BMI ≥ 30 kg/m^2^) in groups DS, D, and S were 34.8%, 52.2%, and 17.4%, respectively. There were significant differences between groups D and S in weight (p = 0.012), BMI (p = 0.002), and the presence of obesity (p = 0.002). The three groups were similar in marital status, education, use of anxiolytics and alcohol consumption.

The proportion of patients who were taking antihypertensives was different among groups DS (58.7%), D (71.7%), and S (30.4%) (p < 0.001). The individuals within the DS group used antihypertensives 3.2 times more than the S group (OR: 3.2481; 95% CI: 1.38 to 7.67). Similarly, the proportion of individuals who exercised at least two times per week was different among groups DS (32.6%), D (58.7%), and S (34.8%) (p = 0.019), and the OR of group D compared to group DS was 2.937 (95% CI: 1.25 to 6.88).

Most of the diabetic patients in the D and DS groups were only taking oral antidiabetic drugs (50% and 61%, respectively, p = 0.917).

There was no difference in the proportion of individuals with symptoms of depression or anxiety in the DS group compared to groups D and S, and their scores on subscales HAD-A and HAD-D were also similar (Table 1). When only men were considered in the analysis, those of D group were 85% less likely to exhibit symptoms of anxiety (OR: 0.1481; 95% CI: 0.03 to 0.79) and 8 times more likely to practice physical activity twice or more per week than men of the DS group (OR: 8.028; 95% CI: 2.15 to 29.94). These results suggest that the lower frequency of anxiety symptoms could have been due to physical activity. Therefore, multiple logistic regression was performed, and we observed that the frequency of physical activity among men was not associated with the prevalence of anxiety symptoms (p = 0.328). There were no significant differences among the three groups of men in the use of anxiolytics, the consumption of alcohol, or the presence of obesity.

**Table 1:** Prevalence of symptoms of anxiety and depression in diabetic smokers (DS), diabetic non-smokers (D), and non-diabetic smokers (S)

**HAD scale**	**DS (n = 46)**	**D (n = 46)**	**S (n = 46)**	**p-value**
HAD-A score (average ± SD)	7.98 ± 4.07	7.37 ± 5.14	8.80 ± 4.58	0.330 *
HAD-A score ≥ 8 (n/%)	23 (50.0%)	18 (39.1%)	29 (63.0%)	0.072 ^#^
· Men	9 (19.6%)	2 (4.3%)	11 (23.9%)	0.011 ^#^
· Women	14 (30.4%)	16 (34.8%)	18 (39.1%)	0.440 ^#^
HAD-D score (average ± SD)	6.17 ± 3.58	6.74 ± 4.60	5.74 ± 4.18	0.516 *
HAD-D score ≥ 8 (n/%)	14 (30.4%)	18 (39.1%)	15 (32.6%)	0.657 ^#^
· Men	5 (10.9%)	6 (13%)	5 (10.9%)	0.922 ^#^
· Women	9 (19.5%)	12 (26.1%)	10 (21.7%)	0.664 ^#^

*Analysis of variance; # chi-squared test.

The proportion of participants who showed symptoms of anxiety (50.7%) was higher than those who had symptoms of depression (34.1%) (p = 0.007), whereas 31.6% had both symptoms. The mean value obtained on the anxiety scale (8.05 ± 4.62) was also higher than the depression value (6.22 ± 4.13) (p < 0.001).

There was a moderate positive linear correlation between depression and anxiety scores (Pearson r = 0.5297; 95% CI: 0.40 to 0.64; p < 0.001). Based on these results, a patient with symptoms of depression (HAD-D subscale score ≥ 8) was 6.5 times more likely to report symptoms of anxiety than those who did not have symptoms of depression (OR: 6.5030; 95% CI: 2.87 to 14.75; p < 0.001) and vice versa.

The daily use of cigarettes, the average time of addiction, the degrees of nicotine dependence measured according to the FTND questionnaire, and the proportion of smokers with high nicotine dependence were all statistically similar between the S and DS groups (Table 2).

**Table 2:** Smoking-related characteristics in diabetic smokers (DS) and non-diabetic smokers (S)

**Characteristics**	**DS (n = 46)**	**S (n = 46)**	**p-value**
**Daily consumption (cigarettes/day)**	20 (10–21.5)	15 (10–20)	0.370 *
** · Men**	20 (15–30)	20 (10–20)	0.925 *
** · Women**	10 (9–20)	15 (10–20)	0.820 *
**Time of smoking (years)**	36.74 ± 14.61	34.26 ± 14.80	0.573 #
**Fagerström score**	4.98 ± 2.50	5.04 ±1.58	0.881 #
**Nicotine dependence**			
** · High**	18 (39.1%)	17 (37.0%)	0.999 ^+^
** · Medium or Low**	28 (60.9%)	29 (63.0%)	

Data are median (25th percentile–75th percentile), average ± SD, or absolute frequency (relative in %).

* Mann–Whitney *U*-test; # Student’s *t* test; ^+^ chi-squared test.

Fagerström scores showed no significant correlation with the scores obtained on the subscale of anxiety (p = 0,735) or depression (p = 0,364). The nicotine-dependent patients had an average anxiety score of 8.51 ± 4.53, while non-nicotine-dependent patients scored 8.32 ± 4.25 (p = 0.832). The mean depression scores were also similar between the two groups (6.46 ± 4.01 and 5.65 ± 3.80, respectively, p = 0.335). Thus, the prevalence of symptoms of anxiety and depression were similar among nicotine dependents (54,3% and 37.1%, respectively) and non-dependents (57,9% and 28.1%, respectively).

Smokers, with and without diabetes, were similar to non-smokers in terms of alcohol consumption (p = 0.122) but were different in terms of physical activity (p = 0.009). The likelihood that an individual would practice physical activity at least twice per week was almost 3 times higher if the individual did not smoke (OR: 2.797; 95% CI: 1.35 to 5.8).

The proportion of men with high nicotine dependence (43.5%) was similar to women (32.6%) (p = 0,284). However, the daily cigarette consumption was higher among men than women (p = 0.018). Thus, the average Fagerström score obtained for the men (5.52 ± 1.96 points) was higher than for women (4.50 ± 2.08 points) (p = 0.002).

Marital status, obesity, use of anxiolytic or antihypertensive drugs, treatment of T2D, family history of smoking, habit of smoking, nicotine dependence, length of education, and physical activity were not associated with the prevalence of anxiety or depression symptoms (Table 3). There was no association between daily or weekly consumption of alcohol and symptoms of depression or nicotine dependence. However, patients who consumed alcohol daily or weekly were 65% less likely to present symptoms of anxiety (OR: 0.35; 95% CI: 0.15 to 0.81). Because 69% of these patients were men, multiple logistic regression was applied, which indicated that alcohol consumption was not significantly associated with the absence of symptoms of anxiety (p = 0.111), but it was associated with male gender (p < .001). There was no association between gender and the habit of practicing physical activity (p = 0.612) or duration of education (p = 0.999). Women showed almost 5 times the likelihood of men to present the symptoms of anxiety and 2.7 times the likelihood of men to present symptoms of depression.

**Table 3:** Analysis of factors associated with anxiety and depression among the study participants

**Variables**	**n**	**HAD-A ≥ 8 score**	**p-value**	**OR # (95% CI)**	**HAD-D ≥ 8 score**	**p-value**	**OR # (CI 95%)**
		**n (%)**			**n (%)**		
**Marital status**							
Non-married	42	24 (57.1)	0.320	1.0	13 (31.0)	0.611	1.0
Married	96	46 (47.9)		0.69 (0.33 to 1.43)	34 (35.4)		1.22 (0.56 to 2.66)
**Obesity (BMI ≥ 30)**							
No	90	43 (47.8)	0.344	1.0	32 (35.6)	0.611	1.0
Yes	48	27 (56.3)		1.41 (0.69 to 2.84)	15 (31.3)		0.82 (0.39 to 1.74)
**Use of anxiolytics**							
No	123	61 (49.6)	0.290	1.0	40 (32.5)	0.192	1.0
Yes	15	9 (60.0)		1.86 (0.59 to 5.86)	7 (46.7)		2.10 (0.69 to 6.39)
**Use of antihypertensives**	64	33 (51.6)	0.855	1.0	18 (28.1)	0.173	1.0
No	74	37 (50.0)		0.94 (0.48 to 1.83)	29 (39.2)		1.65 (0.8 to 3.37)
Yes							
Treatment of T2D*							
With insulin	63	28 (44.4)	0.973	1.0	23 (36.5)	0.609	1.0
Without insulin	29	13 (44.8)		1.02 (0.42 to 2.46)	9 (31.0)		0.78 (0.31 to 2.0)
**Smoking family history**							
Non-smoking parents	33	12 (36.4)	0.062	1.0	11 (33.3)	0.920	1.0
Mother, Father, or both smoked	105	58 (55.2)		2.16 (0.96 to 4.84)	36 (34.3)		1.04 (0.46 to 2.39)
**Smoking habit**							
Non-smokers	46	18 (39.1)	0.056	1.0	18 (39.1)	0.375	1.0
Smokers	92	52 (56.5)		2.02 (0.98 to 4.16)	29 (31.5)		0.72 (0.34 to 1.50)
Nicotine dependence**							
Low or medium	57	33 (57.9)	0.735	1.0	16 (28.1)	0.364	1.0
High (FTND score ≥ 6)	35	19 (54.3)		0.86 (0.37 to 2.02)	13 (37.1)		1.51 (0.62 to 3.71)
**Alcohol consumption:**							
No or ≤ monthly	106	60 (56.6)	**0.014**	1.0	39 (36.8)	0.221	1.0
Daily or weekly	32	10 (31.2)		0.35 (0.15 to 0.81)	8 (25.0)		0.57 (0.23 to 1.40)
**Length of schooling:**							
< 8 years	82	45 (54.9)	0.239	1.0	24 (29.3)	0.152	1.0
≥ 8 years	56	25 (44.6)		0.66 (0.33 to 1.31)	23 (41.1)		1.68 (0.82 to 3.44)
**Gender:**							
Male	69	22 (31.9)	**< 0.001**	1.0	16 (23.2)	**0.008**	1.0
Female	69	48 (69.6)		4.88 (2.38 to 10.04)	31 (44.9)		2.70 (1.30 to 5.63)
**Physical Activity**							
No or ≤ 1x/week	80	45 (56.2)	0.129	1.0	25 (31.2)	0.414	1.0
From 2 to 5x/week	58	25 (43.1)		0.59 (0.30 to 1.17)	22 (37.9)		1.34 (0.66 to 2.74)

* Diabetic patients. ** Smoking patients # Logistic regression.

Marital status, gender, obesity, use of anxiolytic or antihypertensive drugs, physical activity, family history of smoking, and alcohol consumption showed no association with the degree of nicotine dependence (Table 4). Participants who had ≥ 8 years of formal education were 2.57 times more likely to have high nicotine dependence than those with less education.

**Table 4:** Analysis of factors associated with nicotine dependence among smokers

**Variable**	**n**	**Fagerström score ≥ 6**	p-value*	OR* (95% CI)
		**n (%)**		
**Gender:**				
Male	46	20 (43.5)	0.284	1.0
Female	46	15 (32.6)		0.63 (0.27 to 1.47)
**Marital status**				
Non-married	29	15 (51.7)	0.070	1.0
Married	63	20 (31.7)		0.43 (0.18 to 1.07)
**Obesity (BMI ≥ 30)**				
No	68	26 (38.2)	0.949	1.0
Yes	24	9 (37.5)		0.97 (0.37 to 2.53)
**Use of anxiolytics**				
No	78	30 (38.5)	0.973	1.0
Yes	14	5 (35.7)		1.02 (0.31 to 3.41)
**Use of antihypertensives**				
No	51	21 (41.2)	0.491	1.0
Yes	41	14 (34.1)		0.74 (0.32 to 1.74)
**Physical activity**				
No or ≤ 1x/week	61	26 (42.6)	0.207	1.0
From 2 to 5x/week	31	9 (29.0)		0.55 (0.22 to 1.39)
**Smoking family history:**				
Non-smoking parents	16	7 (43.7)	0.606	1.0
Mother, Father, or both smoked	76	28 (36.8)		0.75 (0.25 to 2.24)
**Alcohol consumption:**				
No or ≤ monthly	67	22 (32.8)	0.096	1.0
Daily or weekly	25	13 (52.0)		2.22 (0.87 to 5.65)
**Length of schooling:**				
< 8 years	55	16 (29.1)	**0.033**	1.0
≥ 8 years	37	19 (51.4)		2.57 (1.08 to 6.13)

*Logistic regression.

## Discussion

In this study the prevalence of symptoms of depression and anxiety in smokers with T2D was 30.4% and 50%, respectively. There was no difference between the proportion of individuals with symptoms of depression or anxiety in the DS group and that of group D or S. Among male patients with T2D, the smokers had a higher prevalence of anxiety symptoms than the non-smokers. Finally, the degree of nicotine dependence showed no correlation with the scores obtained on the subscale of anxiety or depression.

Although the similar prevalence of depression among people with diabetes who are smokers or non-smokers was suggested by Lloyd *et al.*[15], other studies showed a higher prevalence among smokers [12,14,31]. Unlike the present study, none of the previously published studies evaluated only patients with T2D.

Epidemiological studies have found heterogeneity among the prevalence of depression over 1 year and throughout life in the general population. A systematic review, using a pooled sample of studies, found a 1-year prevalence of 4.1% and a lifetime prevalence of 6.7% [32]. Other studies show that the prevalence of depressive disorders in the general population ranges from 6.3 to 6.6% (annual) and 8.3 to 16.2% (lifetime) [33,34]. However, Haug *et al.*[35], using the same methods as this study (HAD scale), found a prevalence of 10.4% for symptoms of depression in a sample of 62,651 individuals from the Norwegian general population.

In Brazil, although it has been evaluated in different places, in different sub-populations, and in different ways, population-based studies have found a prevalence of depression throughout life ranging from 2 to 16.8% [36,37] and an annual rate of 7.1% [37]. Moreover, the prevalence of depressive symptoms varies among different subgroups of the general Brazilian population, for example, 24.4% in individuals with epilepsy [38], 14 to 37% in hospitalized patients [39,40] and 8,5% among college students [41].

Two meta-analyses evaluating depression in the population with diabetes in relation to the general population have been published. The prevalence of depression is higher in diabetics compared to non-diabetics [22], and the risk of depression is 24% higher in patients with T2D [23]. The prevalence of depression in T2D patients varies from 17.9 to 43.5% [24-26]. Thus, the high prevalence of symptoms of depression in groups DS, D, and S of this study may be explained by its association with diabetes [21-24,26], which is a chronic disease, and with smoking, which is a harmful behavior to health and is also associated with depression [12,14,21].

Regarding the frequency of anxiety symptoms in the general population, Bourdon *et al.*[33] estimated the prevalence of anxiety disorders in the general population of the U.S. at 10.1% (annual) and 14.6% (lifetime). Two international studies that used the HAD scale found a prevalence of 15.3% in a sample of 62,651 individuals in Norway [35] and 15% in 2061 normoglycemic individuals in the Netherlands [26]. In Brazil, the prevalence of any anxiety disorder ranges from 9.6 to 12.5% (lifetime) [36,37], 7.7% (annual) [37], 6% (monthly) [37], and 36.2% (one time) [42]. Although it is difficult to compare because the data collection methods differ, but it is clear that the prevalence of anxiety varies widely. Among specific groups, different prevalence values have been found: 16.5% among college students [41], 39.4% among epileptics [38], and 46% in hospitalized patients [40].

Anxiety is associated with smoking [12] and nicotine dependence [11,13]. Despite the limited literature on the prevalence of anxiety in smokers with diabetes, some studies show a higher prevalence of anxiety symptoms among subjects with diabetes smokers than non-smokers [14,15]. In the diabetic population, several studies show that the prevalence of anxiety varies from 19.9 to 57.9% [12,15,25,26]. A systematic review found that generalized anxiety disorders are present in 14% and anxiety symptoms in approximately 40% of people with diabetes. The prevalence was similar among individuals with T1D and T2D (41.3% vs. 42.2%, p = 0.80) [43]. Thus, the similar prevalence of anxiety symptoms found in the three groups of this study, which were higher than the rates found in the general population, can be explained by its association with diabetes [12,21,25] and smoking [12,21].

In a study examining the psychometric properties of the HAD scale at the population level, the authors found a HAD-A subscale score of 4.06 and HAD-D subscale score of 3.87 [44]. According to the present study, the average scores of 8.05 for anxiety and 6.22 for depression suggest a higher prevalence of symptoms of anxiety and depression in the sample studied when compared to the general population. Our averages are closer to the values found in hospitalized patients reported in the study of Delfini *et al.*[40], which found mean scores of 7.9 on HAD-A and 6.92 on HAD-D.

The results of this study reinforce the results reported by other authors that show a positive linear correlation between the scores of anxiety and depression [45] and a higher prevalence of depression and/or anxiety disorders in women than in men [11,21,25,40,43].

A survey conducted by the German Ministry of Science found a 9.4% annual prevalence of nicotine dependence in a sample of approximately 3,300 individuals in the non-institutionalized population of Germany. Among 1491 regular smokers in the study, the prevalence was 26.2% [46]. In the U.S., based on a sample of 4,414 smokers, Breslau *et al.*[28] estimated the prevalence of nicotine dependence to be 24%. In Brazil, a study conducted in Rio Grande do Sul in the general population of smokers without tobacco-related diseases founds 13.3% of high nicotine dependence [11]. In another study involving hospitalized patients, 21% were regular smokers, and among these, 26% had high nicotine dependence [47].

The presence of nicotine dependence varies according to age [28,37]. Breslau *et al.*[13] did not identify any smokers with high nicotine dependence, which is likely due to the youth of their sample (average age 26 years). The study of Castro *et al.*[11] observed high nicotine dependence in 13.3% of smokers, whose average age was 37.7 years. In another study involving 573 elderly (≥ 60 years) admitted to long-term institutions in Brazil, 25.9% of the smokers had high nicotine dependence [48]. Therefore, the high percentages of nicotine dependence among smokers with diabetes (39.1%) and non-diabetics (38.5%) of the present study (mean age 53 years) may have been due in part to age.

Fagerström and Furberg [49] compared 15 studies conducted in 13 different countries that used FTND, and they found that scores ranged from 2.8 to 4.6 (average 3.6). Germany and Norway had the lowest scores, the U.S. and Sweden had the highest. The results from the latter two countries resemble those of the present study because the average FTND scores in both groups of smokers, with and without diabetes, were approximately 5 points.

The high rates of anxiety and depression among nicotine dependents in the present study (54.3% and 37.1%, respectively) are in accordance with the prevalences reported in the literature: 22 to 62.3% for any anxiety disorder and 16.6 to 39% for major depression [13,50]. However, the lack of association between nicotine dependence and symptoms of anxiety and depression differs from other studies [11,13]. There is no clear explanation for this difference, but the studies involved different types of subjects and therefore would not necessarily achieve the same results. While two studies [11,13] evaluated the general population of smokers, in this study the sample was composed of outpatients. Moreover, the non-nicotine-dependent individuals of the DS group were all diabetic, so the presence of T2D could have influenced the prevalence of symptoms.

Interestingly, this study found a negative association of smoking, disregarding the presence or absence of diabetes or nicotine dependence, with physical activity. Among men, smoking was also associated with a higher prevalence of anxiety symptoms. The interactions among all of these variables are clearly complex, but symptoms of depression and anxiety have been strongly associated with some adverse health behaviors, such as smoking, alcohol consumption, and physical inactivity [12,21,25]. Therefore, these behaviors are likely interrelated. For example, smoking is associated with low physical activity, a diet low in fruits and vegetables, and high alcohol consumption [3]. Given that physical activity promotes better blood glucose control, lipid profile, and blood pressure in patients with T2D [51] and that smoking is a risk factor for macro- and microvascular complications in individuals with diabetes [9,10,18], the results of the present study reinforce the importance of smoking cessation for smokers T2D.

Unlike the present study, Breslau *et al.*[13] observed that the amount of education was inversely related to the degree of nicotine dependence. It has not been possible to find a convincing explanation for this difference. Khuwaja *et al.*[25] identified hypertension as an independent factor associated with symptoms of anxiety and depression, but the present study showed no such association. This may have been due to the small number of individuals evaluated in this regard. The higher proportion of smokers with diabetes who used antihypertensives when compared to smokers without diabetes was likely due to the diabetes affects the heart and blood vessels. Cardiovascular disease is the most common cause of death and disability among subjects with diabetes [4].

If there are differences in the prevalence of symptoms of depression and anxiety between smokers with and without T2D, it is likely that those differences are very little. Thus, we wish to emphasize that this study has limitations because of the small number of patients studied and the possible type II errors. However, the only existing study aiming to assess depression and anxiety in diabetic smokers recruited only 83 patients with type 1 diabetes and only 19 of these were smokers [14].

## Conclusions

The prevalence of depression and anxiety among smokers with and without T2D and non-smokers T2D is similar. Among male individuals with T2D, the smokers have more symptoms of anxiety than the non-smokers. There is no difference in the prevalence of nicotine dependence between smokers with and without diabetes. The presence of symptoms of anxiety or depression is similar between patients who are dependent and not dependent on nicotine.

## Competing interests

No potential conflicts of interest relevant to this article were reported.

## Authors’ contributions

SFO approached the patients. He also worked on the acquisition, tabulation, and analysis of data and writing of the manuscript. PTJ, MTJ, and MLMPJ participated in designing the study, analyzing and interpreting the results, preparing the manuscript, and critically reviewing the intellectual content. RMCP participated in the statistical analysis. JSA trained students for the application of the HAD scale. MTJ and LDPMF participated in the approach to patients and applying the HAD scale. All authors read and approved the final manuscript.

## Grant support

No grant support to this article was reported.
